# Supplementary material for: Boundary conditions investigation to improve computer simulation of cerebrospinal fluid dynamics in hydrocephalus patients
Source: Commun Biol. 2021 Mar 23;4:394. doi: 10.1038/s42003-021-01920-w (PMC7988041; doi:10.1038/s42003-021-01920-w)
Supplement: Supplementary file 2 — Description of Additional Supplementary Files [file 42003_2021_1920_MOESM2_ESM.pdf]

Description of Additional Supplementary Files

File name: Supplementary Data 1

Description: Raw data for Fig. 1a; source data for maximum aqueductal CSF stroke volume for healthy subjects and patients under BCs "A", "B", and "C".

File name: Supplementary Data 2

Description: Raw data for Figs. 3a-c; source data for the simulation data and CINE PC-MRI data of the maximum CSF velocity in the CA for healthy subjects and patients under BCs "A", "B", and "C".
