# Supplementary material for: Boundary conditions investigation to improve computer simulation of cerebrospinal fluid dynamics in hydrocephalus patients
Source: Commun Biol. 2021 Mar 23;4:394. doi: 10.1038/s42003-021-01920-w (PMC7988041; doi:10.1038/s42003-021-01920-w)
Supplement: Supplementary file 5 — Reporting Summary [file 42003_2021_1920_MOESM5_ESM.pdf]

nature research

Corresponding author(s): Seifollah Gholampour
Last updated by author(s): Feb 17, 2021

Reporting Summary

Nature Research wishes to improve the reproducibility of the work that we publish. This form provides structure for consistency and transparency in reporting. For further information on Nature Research policies, see our Editorial Policies and the Editorial Policy Checklist.

Statistics

For all statistical analyses, confirm that the following items are present in the figure legend, table legend, main text, or Methods section.

n/a	Confirmed	
☐	☒	The exact sample size (n) for each experimental group/condition, given as a discrete number and unit of measurement
☐	☒	A statement on whether measurements were taken from distinct samples or whether the same sample was measured repeatedly
☐	☒	The statistical test(s) used AND whether they are one- or two-sided<br>*Only common tests should be described solely by name; describe more complex techniques in the Methods section.*
☐	☒	A description of all covariates tested
☐	☒	A description of any assumptions or corrections, such as tests of normality and adjustment for multiple comparisons
☐	☒	A full description of the statistical parameters including central tendency (e.g. means) or other basic estimates (e.g. regression coefficient) AND variation (e.g. standard deviation) or associated estimates of uncertainty (e.g. confidence intervals)
☐	☒	For null hypothesis testing, the test statistic (e.g. F, t, r) with confidence intervals, effect sizes, degrees of freedom and P value noted<br>*Give P values as exact values whenever suitable.*
☐	☒	For Bayesian analysis, information on the choice of priors and Markov chain Monte Carlo settings
☐	☒	For hierarchical and complex designs, identification of the appropriate level for tests and full reporting of outcomes
☐	☒	Estimates of effect sizes (e.g. Cohen's d, Pearson's r), indicating how they were calculated

Our web collection on statistics for biologists contains articles on many of the points above.

Software and code

Policy information about availability of computer code

Data collection	We have used the MRI files of the subjects, and we prepared all of these data according to the ethical standards of the Shohada Tajrish Hospital.
Data analysis	We have used ADINA 8.3 (Adina R&D Inc., Watertown MA, USA) software for data analysis. However, we used the routine toolbar of this software and we didn't use any specific codes.

For manuscripts utilizing custom algorithms or software that are central to the research but not yet described in published literature, software must be made available to editors and reviewers. We strongly encourage code deposition in a community repository (e.g. GitHub). See the Nature Research guidelines for submitting code & software for further information.

Data

Policy information about availability of data

All manuscripts must include a data availability statement. This statement should provide the following information, where applicable:

- Accession codes, unique identifiers, or web links for publicly available datasets
- A list of figures that have associated raw data
- A description of any restrictions on data availability

For data validation, we compared the computer simulation data with experimental data of ICP-monitoring and also invivo CSF velocity data. We listed the results of these comparisons in the Table 1 and Fig.1f and g, and also Fig. 3a-c. The MRI files of subjects contain some identifying information of patients and normal subjects, and cannot be made publicly available. All relevant data are available from the corresponding author upon request. Raw data for Fig. 1a and Fig. 3a-c are included in Supplementary Data files 1 and 2, and raw data for Fig. 3d-g is also included in Tables 2 and 3.

Field-specific reporting

Please select the one below that is the best fit for your research. If you are not sure, read the appropriate sections before making your selection.

☒ Life sciences ☐ Behavioural & social sciences ☐ Ecological, evolutionary & environmental sciences

For a reference copy of the document with all sections, see nature.com/documents/nr-reporting-summary-flat.pdf

Life sciences study design

All studies must disclose on these points even when the disclosure is negative.

Sample size	eight healthy subjects and 11 hydrocephalus patients
Data exclusions	(N/A): no data were excluded
Replication	All attempt at replication were successful
Randomization	Allocation of subjects was random
Blinding	Prior to scanning, written informed consent was obtained from all the volunteers. All MRI data were anonymized prior to transfer to operators for analysis.

Reporting for specific materials, systems and methods

We require information from authors about some types of materials, experimental systems and methods used in many studies. Here, indicate whether each material, system or method listed is relevant to your study. If you are not sure if a list item applies to your research, read the appropriate section before selecting a response.

Materials & experimental systems

n/a	Involved in the study	
☐	☐	Antibodies
☐	☐	Eukaryotic cell lines
☐	☐	Palaeontology and archaeology
☐	☐	Animals and other organisms
☐	☒	Human research participants
☐	☒	Clinical data
☐	☐	Dual use research of concern

Methods

n/a	Involved in the study	
☐	☐	ChIP-seq
☐	☐	Flow cytometry
☐	☒	MRI-based neuroimaging

Antibodies

Antibodies used	(N/A): We did not use any antibody
Validation	We had data validation section, however, we did not use any antibody

Eukaryotic cell lines

Policy information about cell lines

Cell line source(s)	(N/A): We did not use any cell line source
Authentication	(N/A): We did not use any cell line source
Mycoplasma contamination	(N/A): We did not use any cell line source
Commonly misidentified lines (See ICLAC register)	(N/A): We did not use any cell line source

Palaeontology and Archaeology

Specimen provenance	(N/A)

Specimen deposition | (N/A)

Dating methods | (N/A): No new dates are provide

☐ Tick this box to confirm that the raw and calibrated dates are available in the paper or in Supplementary Information.

Ethics oversight | *Identify the organization(s) that approved or provided guidance on the study protocol, OR state that no ethical approval or guidance was required and explain why not.*

Note that full information on the approval of the study protocol must also be provided in the manuscript.

Animals and other organisms

Policy information about studies involving animals; ARRIVE guidelines recommended for reporting animal research

Laboratory animals	(N/A): This study did not involve laboratory animals
Wild animals	(N/A): This study did not involve laboratory animals
Field-collected samples	(N/A): This study did not involve laboratory animals
Ethics oversight	(N/A): This study did not involve laboratory animals

Note that full information on the approval of the study protocol must also be provided in the manuscript.

Human research participants

Policy information about studies involving human research participants

Population characteristics	11 hydrocephalus patients and eight healthy subjects were recruited. The age range of the patients (six males and five females) and the healthy subjects (four males and four females) was 53-74 and 48-69 years, respectively. Moreover, the range of the body mass index of the patients and the healthy subjects was 23.8-29.4 and 25.0-30.2, respectively. Shohada Tajrish Neurosurgical team diagnose and treated these patients.
Recruitment	11 patients and eight healthy subjects were recruited from a total of 42 hydrocephalus patients and 23 healthy subjects based on their age range and BMI.
Ethics oversight	the data access and ethics committees of Shohada Tajrish Neurosurgical Centre of Excellence.

Note that full information on the approval of the study protocol must also be provided in the manuscript.

Clinical data

Policy information about clinical studies

All manuscripts should comply with the ICMJE guidelines for publication of clinical research and a completed CONSORT checklist must be included with all submissions.

Clinical trial registration	(N/A): This study was not Clinical trial
Study protocol	(N/A): This study was not Clinical trial
Data collection	(N/A): This study was not Clinical trial
Outcomes	(N/A): This study was not Clinical trial

Dual use research of concern

Policy information about dual use research of concern

Hazards

Could the accidental, deliberate or reckless misuse of agents or technologies generated in the work, or the application of information presented in the manuscript, pose a threat to:

No	Yes	
☒	☐	Public health
☒	☐	National security
☒	☐	Crops and/or livestock
☒	☐	Ecosystems
☒	☐	Any other significant area

Experiments of concern

Does the work involve any of these experiments of concern:

No	Yes	
☒	☐	Demonstrate how to render a vaccine ineffective
☒	☐	Confer resistance to therapeutically useful antibiotics or antiviral agents
☒	☐	Enhance the virulence of a pathogen or render a nonpathogen virulent
☒	☐	Increase transmissibility of a pathogen
☒	☐	Alter the host range of a pathogen
☒	☐	Enable evasion of diagnostic/detection modalities
☒	☐	Enable the weaponization of a biological agent or toxin
☒	☐	Any other potentially harmful combination of experiments and agents

ChIP-seq

Data deposition

☐ Confirm that both raw and final processed data have been deposited in a public database such as GEO.

☒ Confirm that you have deposited or provided access to graph files (e.g. BED files) for the called peaks.

Data access links<br>*May remain private before publication.*	We create our data base with regards to all policy of our ethical approval and data access committee. According to this policy for accessing the data should contact with corresponding author
Files in database submission	We create our data base with regards to all policy of our ethical approval and data access committee. According to this policy for accessing the data should contact with corresponding author
Genome browser session<br>(e.g. UCSC)	We create our data base with regards to all policy of our ethical approval and data access committee. According to this policy for accessing the data should contact with corresponding author

Methodology

Replicates	First, the CSF velocity diagram in CA calculated from the FSI simulation of each healthy subject and each patient was compared with the CSF velocity diagram in CA which was measured experimentally using the CINE phase-contrast magnetic resonance imaging (CINE PC-MRI) of them. Second, the values of CSF pressure in SAS were experimentally measured in 10 of the 11 patients by ICP monitoring were compared with CSF pressure calculated from the FSI simulation. All experimental test were repeated five times.
Sequencing depth	All experimental test were repeated five times. It should be noted the time and length of tests were not important and effective in the tests.
Antibodies	(N/A): No antibodies were used in the tests.
Peak calling parameters	(N/A): No command line program and ... were used in the tests.
Data quality	For insurance about correctness of our data we have compared our computer simulation data with the experimental data (CINE PC-MRI and ICP monitoring).
Software	DICOM files obtained from MRI of each healthy subject and patient were transferred to Mimics software v13.1 to prepare the points cloud that the points in the cloud were voxel centres. The point clouds of the head substructures (SAS, brain tissue, and ventricular system) were produced for each healthy subject and patient and transferred to CATIA v5.R21 for 3D geometrical modeling. After creating 3D geometrical models of the head of the healthy subjects and patients separately (Fig. 4d), the models were transferred to ADINA 8.3 (Adina R&D Inc., Watertown MA, USA) for meshing (Fig. 4e) and analysis. It should be noted that we only used the routine toolbars of these softwares and we did not write any programming.

Flow Cytometry

Plots

Confirm that:

☐ The axis labels state the marker and fluorochrome used (e.g. CD4-FITC).

☒ The axis scales are clearly visible. Include numbers along axes only for bottom left plot of group (a 'group' is an analysis of identical markers).

☒ All plots are contour plots with outliers or pseudocolor plots.

☐ A numerical value for number of cells or percentage (with statistics) is provided.

Methodology

Sample preparation: Accordingly, 11 patients and eight healthy subjects were recruited.

Instrument: MRI

Software: DICOM files obtained from MRI of each healthy subject and patient were transferred to Mimics software v13.1 to prepare the points cloud that the points in the cloud were voxel centres. The point clouds of the head substructures (SAS, brain tissue, and ventricular system) were produced for each healthy subject and patient and transferred to CATIA v5.R21 for 3D geometrical modeling. After creating 3D geometrical models of the head of the healthy subjects and patients separately (Fig. 4d), the models were transferred to ADINA 8.3 (Adina R&D Inc., Watertown MA, USA) for meshing (Fig. 4e) and analysis. It should be noted that we only used the routine toolbars of these softwares and we did not write any programming.

Cell population abundance: (N/A): We did not use Cell in our study

Gating strategy: (N/A): We did not use Cell in our study

☒ Tick this box to confirm that a figure exemplifying the gating strategy is provided in the Supplementary Information.

Magnetic resonance imaging

Experimental design

Design type: We used event-relevant

Design specifications: Each tests were repeated for 5 times. Scanning was performed using a 3 Tesla MRI system (Magnetom Trio, Siemens, Erlangen, Germany), with the acquisition time of 45 minutes.

Behavioral performance measures: Each tests were repeated for 5 times. We calculated the mean, SD, confidence interval and coefficient of variation.

Acquisition

Imaging type(s): We used structural imaging

Field strength: Scanning was performed using a 3 Tesla MRI system (Magnetom Trio, Siemens, Erlangen, Germany)

Sequence & imaging parameters: A velocity encoding value (VENC) of 100 cm/s was chosen to measure the CSF flow. Further parameters used in the measurement included: repetition time = 18 msec; flip angle = 23°; echo time = 8.3 msec; field of view = 23 cm; slice thickness = 3 mm; and matrix size = 256×198. The pixel velocity in CSF areas was corrected by subtracting the average velocity of solid brain tissue in a nearly 29×29 mm2 area surrounding the pixel.

Area of acquisition: The pixel velocity in CSF areas was corrected by subtracting the average velocity of solid brain tissue in a nearly 29×29 mm2 area surrounding the pixel.

Diffusion MRI ☐ Used ☒ Not used

Preprocessing

Preprocessing software: DICOM files obtained from MRI of each healthy subject and patient were transferred to Mimics software v13.1 to prepare the points cloud that the points in the cloud were voxel centres. The point clouds of the head substructures (SAS, brain tissue, and ventricular system) were produced for each healthy subject and patient and transferred to CATIA v5.R21 for 3D geometrical modeling. After creating 3D geometrical models of the head of the healthy subjects and patients separately (Fig. 4d), the models were transferred to ADINA 8.3 (Adina R&D Inc., Watertown MA, USA) for meshing (Fig. 4e) and analysis. It should be noted that we only used the routine toolbars of these softwares and we did not write any programming.

Normalization: The inlet of BC "B" and inlet and outlets of BC "C" were calculated with the superposition of a constant value of flow rate and the normalized pulsatile profile of the blood flow rate in the basilar artery, which was measured with the CINE PC-MRI for all the healthy subjects and hydrocephalus patients. The process of normalization were calculated using MATLAB software.

Normalization template: We only used the MATLAB toolbars for normalizing the flow rate function of the blood for inlet inlet of BC "B" and inlet and outlets of BC "C".

Noise and artifact removal: (N/A): We did not use the noise removing process.

Volume censoring: (N/A): We did not use volume censoring

Statistical modeling & inference

Model type and settings: Pearson correlation coefficient (PCC) was used in the present study to assess the correlation between the maximum CSF pressure in SAS and the ventricular system volume (two effective and accurate indices) under the three inlet/outlet BCs for 11 patients.

Effect(s) tested | (N/A): We have not need to assess the effects test

Specify type of analysis: ☒ Whole brain ☐ ROI-based ☐ Both

Statistic type for inference (See Eklund et al. 2016) | (N/A): We have not need to assess voxel/cluster-wise.

Correction | (N/A): We have not need to assess the correction.

Models & analysis

n/a | Involved in the study
- ☒ | ☐ Functional and/or effective connectivity
- ☐ | ☒ Graph analysis
- ☒ | ☐ Multivariate modeling or predictive analysis

Graph analysis

The results of the Shapiro-Wilk test showed that the datasets had a normal distribution. Parametric ANOVA Multiple Comparison was used [99] to compare the CSF pressure, the aqueductal CSF stroke volume, the Reynolds number, the CSF velocity, and the volume between healthy subjects and patients under the three BCs "A", "B", and "C". The homogeneity of the variance test showed that all the variances were equal. Hence, Tukey's post-hoc test was used for pair-wise comparison after ANOVA when comparing the data under the three BCs. Moreover, the normally distributed data led to using Student's t-test after ANOVA to compare both groups of CSF velocities obtained from computer simulation and CINE PC-MRI, and two groups of CSF pressures obtained from computer simulation and ICP-monitoring for assessing data validation. Student's t-test with equal variance was also used to compare the CSF pressure results of CFD and FSI simulations. The test statistics for ANOVA and Student's t-test were T and F, respectively.
The PCC, a number between -1 and +1, is an index for evaluating the correlation between two phenomena [100]. Hence, after ensuring the normal distribution of the data, PCC was used to assess the correlation between the maximum CSF pressure and the ventricular system volume under the three BCs. The data were described as mean±SE, and the P-value of 0.05 was considered statistically significant.
